# Small non-coding RNA transcriptome of the NCI-60 cell line panel

**DOI:** 10.1038/sdata.2017.157

**Published:** 2017-10-24

**Authors:** Erin A. Marshall, Adam P. Sage, Kevin W. Ng, Victor D. Martinez, Natalie S. Firmino, Kevin L. Bennewith, Wan L. Lam

**Affiliations:** 1 Department of Integrative Oncology, British Columbia Cancer Research Centre, Vancouver, British Columbia, Canada V5Z 1L3

**Keywords:** Cancer genomics, RNA sequencing, Non-coding RNAs

## Abstract

Only 3% of the transcribed human genome is translated into protein, and small non-coding RNAs from these untranslated regions have demonstrated critical roles in transcriptional and translational regulation of proteins. Here, we provide a resource that will facilitate cell line selection for gene expression studies involving sncRNAs in cancer research. As the most accessible and tractable models of tumours, cancer cell lines are widely used to study cancer development and progression. The NCI-60 panel of 59 cancer cell lines was curated to provide common models for drug screening in 9 tissue types; however, its prominence has extended to use in gene regulation, xenograft models, and beyond. Here, we present the complete small non-coding RNA (sncRNA) transcriptomes of these 59 cancer cell lines. Additionally, we examine the abundance and unique sequences of annotated microRNAs (miRNAs), PIWI-interacting RNAs (piRNAs), small nuclear RNAs (snRNAs), and small nucleolar RNAs (snoRNAs), and reveal novel unannotated microRNA sequences.

## Background & Summary

The NCI-60 Human Tumour Cell Lines Screen is an initiative started by the National Institutes of Health (NIH) in the late 1980s, focusing on the development of 59 human tumour cell lines for use as an *in vitro* drug screen model^1–3^ (Table 1 (available online only)). These cell lines, derived from nine solid and blood malignancies, have shown great utility both in its original purpose for therapeutic screening as well as in basic cancer research (reviewed by Shoemaker *et al.*^4^). They have since been extensively characterized for various molecular features, including karyotypic complexity^1^, DNA fingerprinting^2^, gene expression microarray profiling^5,6^, and human leukocyte antigen typing^3^. However, the small non-coding RNA (sncRNA) transcriptomes of the NCI-60 cell lines have yet to have been reported at the sequencing level.

The advent of next-generation sequencing has revealed the large proportion of non-coding genes in the human genome, and the relevance of these non-coding species in regulating the expression of both neighbouring and distant protein-coding genes. In the context of cancer, microRNAs (miRNAs) remain the best-studied non-coding RNA species, and have been implicated in all stages of cancer: initiation, progression, and response to therapy (reviewed by Hayes *et al.*^7^). Recent advances in the bioinformatic tools used for the discovery of small non-coding RNA have considerably expanded the number of known miRNA sequences^8^. Other types of sncRNA, including PIWI-interacting RNAs (piRNAs), small nuclear RNAs (snRNAs), and small nucleolar RNAs (snoRNAs) are emerging topics in cancer biology (reviewed by Ng *et al.* and Mannoor *et al.*^9,10^). Beyond their functions in gene regulation, sncRNAs are attractive prognostic biomarkers due to their abundance and stability in various biofluids^11^.

**Figure 1:**
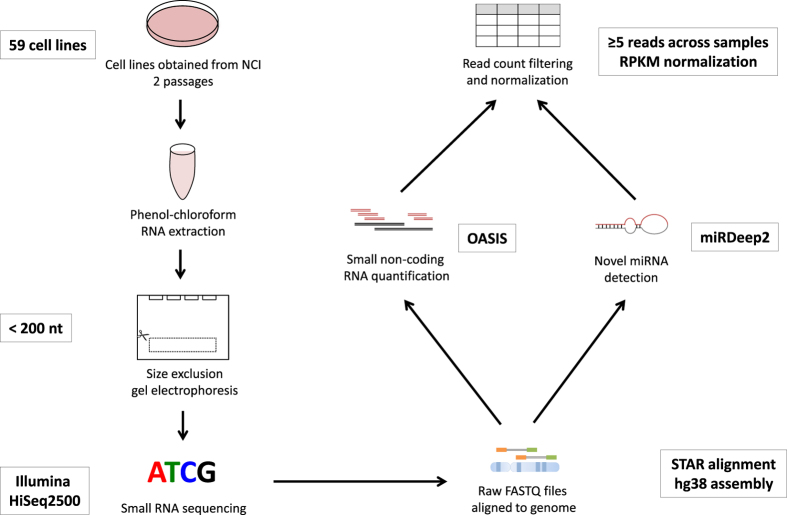
Experimental workflow. Graphical representation of experimental procedure used to extract, process, and analyze RNA from cell lines.

We sequenced the sncRNA transcriptomes of the 59 cell lines in the panel (Fig. 1). SncRNA profiles were generated using the OASIS analysis platform v2.0 (ref. 12). For known sncRNA species (miRNAs, piRNAs, snoRNA, snRNA, and rRNA), high quality reads were mapped to the hg38 build of the human genome and quantified based on annotations containing their specific chromosomal locations. Detection of novel miRNAs was performed using well-established prediction algorithms that assess reads for miRNA folding characteristics, among other factors that indicate the probability that the tested sequence belongs to the miRNA family of sncRNAs^13^. In total, the genomic loci of 49,961 sncRNAs were examined. Using a detection threshold of greater than or equal to 5 reads across all tissues, we detected a total of 24,621 unique sncRNAs [Data Citation 1].

**Figure 2:**
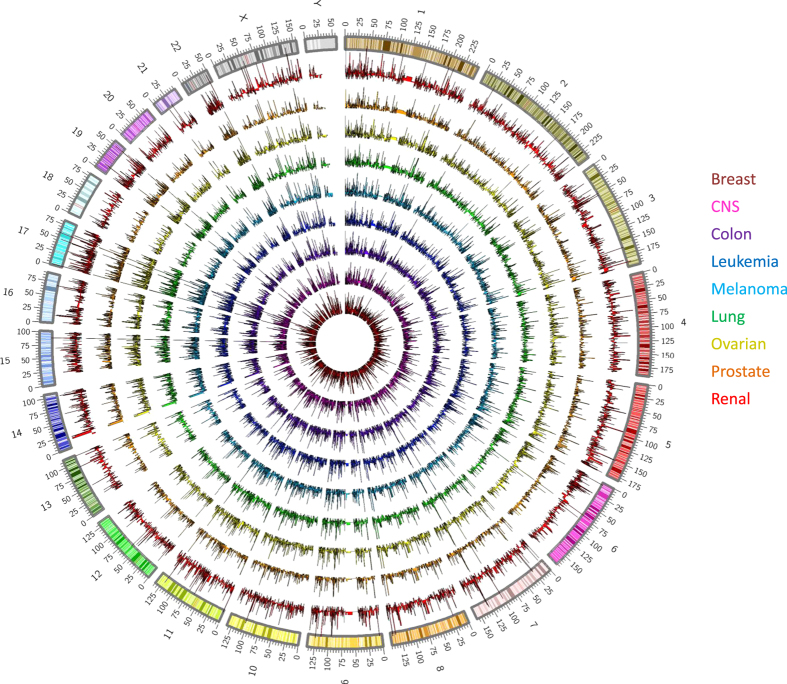
Genome-wide distribution of expressed small non-coding RNA by tissue type. Genomic position of sncRNAs detected (reads≥5) in each tissue type in reference to the hg38 chromosome build karyotype. From inner-most ring to outer: breast (red), CNS (magenta), colon (purple), leukemia (blue), melanoma (teal), lung (green), ovarian (yellow), prostate (orange), and renal (red).

We then examined the genomic distribution of the detected sncRNAs across all tissue types (Table 2,Fig. 2). Notably, sncRNAs are expressed across all chromosomes in every tissue type assessed. SncRNA loci commonly expressed among all tissues may indicate their involvement in preserved biological or cancer-relevant processes, whereas differences in expression may denote tissue specificity.

**Figure 3:**
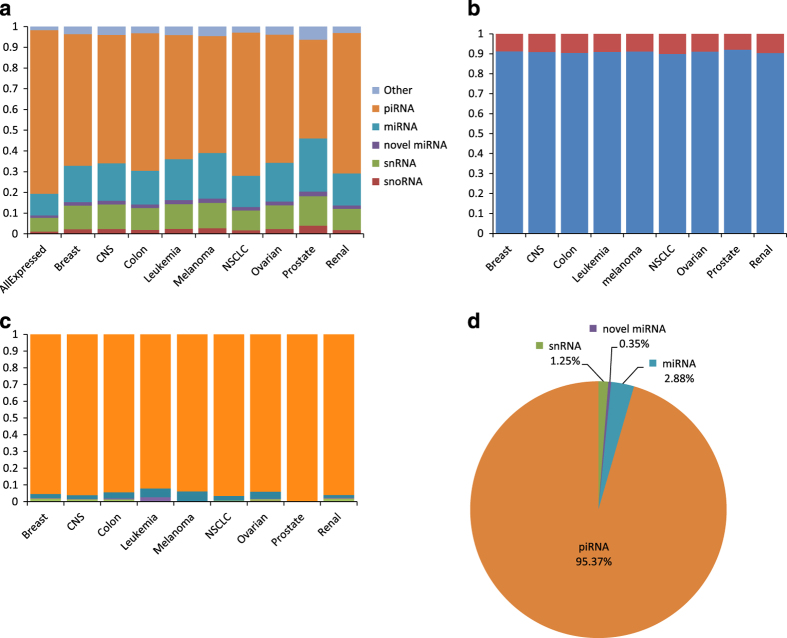
sncRNA distribution by tissue type. (**a**) Relative fraction of sncRNA species detected per tissue type. (**b**) Average fraction of currently annotated (blue) and novel unannotated (red) miRNA per tissue type. (**c**) Relative fraction of tissue-specific unique sncRNA sequences detected per tissue type. (**d**) Fraction of tissue-specific unique sncRNA species.

We also examined the relative frequency of detection for each sncRNA species, both in the entire NCI-60 cell line panel and in lines grouped by organ type (Fig. 3a). Beyond those annotated in miRBase (v.21), novel unannotated miRNAs were determined by integrating secondary structure formation potential with free energy scoring^14^. These novel miRNAs represent an increase of approximately 10% of total miRNAs expressed across all tissue types (Fig. 3b), highlighting the constant expansion of the known non-coding transcriptome as sequencing technologies and bioinformatic tools advance. Consistent with the number of annotated loci in the human genome, piRNAs represent the largest proportion of sncRNA species expressed, followed by miRNA and snRNA (Fig. 3a). Of note, an appreciable number of tissue-specific piRNA sequences across all tissues analyzed increased the relative fraction of piRNAs for all tissues expressed (Fig. 3c,d). Thus, as parts of the small non-coding RNA transcriptome are significantly understudied, we provide this resource to the research community for studying sncRNA-related genetic and epigenetic regulation in cancer using the NCI-60 cell models.

## Methods

### Cell line and sequencing information

Cell line doubling times were obtained directly from the National Institutes of Health NCI (https://dtp.cancer.gov/discovery_development/nci-60/cell_list.htm), and year-of-origin information refers to data of first publication containing the cell line (Table 1 (available online only)). Cell lines were obtained directly from the National Cancer Institute (NCI), were thawed and passaged twice precisely before total RNA was manually extracted using phenol-chloroform protocols from all cell lines using Trizol reagent (Invitrogen, CA, USA). 5,000 ng of extracted RNA per sample was used for sequencing input. Sequencing was performed in accordance with The Cancer Genome Atlas miRNA sequencing protocol (described by Chu *et al.*^15^). Briefly, after ligation to adaptors, 15 cycles of PCR was performed for amplification (98 °C-15 s, 62 °C-30 s and 72 °C-15 s), followed by 5 min at 72 °C. Small RNA exclusion was performed using gel extraction on a 3% MetaPhor Agarose gel (Lonza Inc., Basel, Switzerland), selecting species shorter that 200 nucleotides in order to enrich for targets optimized at 22 nucleotides in length, and was subsequently ethanol-precipitated. Library quality was confirmed by analysis on the Agilent Bioanalyzer DNA1000 chip (Agilent Technologies). Small non-coding RNA sequencing was performed on the Illumina HiSeq2500 platform at the Michael Smith Genome Sciences Centre at the BC Cancer Research Centre, with 8 multiplexed libraries per sequencing lane (Table 3 (available online only), Fig. 1)^15,16^. Data resulting from small non-coding RNA sequencing can be found on the *Sequence Read Archive* [Data Citation 2].

### Pre-processing and small non-coding RNA species detection

**Figure 4:**
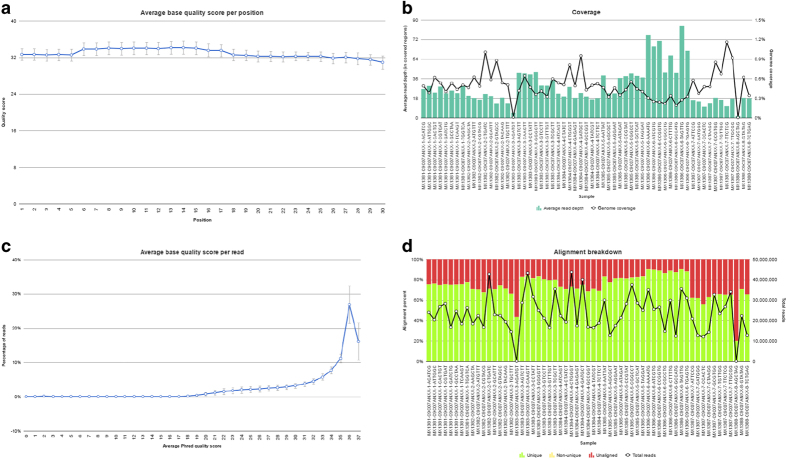
Sequencing and mapping quality. (**a**) Phred quality score per sncRNA base position. (**b**) Genome-wide read depth (column) and genome coverage (line) per sample. (**c**) Fraction of sequencing reads per Phred score. (**d**) Percentage of total reads aligned (unique: green, unaligned: red).

Small-RNA sequencing data was analyzed according to published protocols^17^. In order to extract information for the sncRNA species of interest, unaligned reads (in FASTQ format) were trimmed for adaptors (Cutadapt v1.7.1) and based on sequencing quality (‘trim bases’ from Partek Flow v6.0.17.0614) to reach a Phred quality score ≥20 (Fig. 4a–d). FASTQ files were then aligned using the Spliced Transcripts Alignment to a Reference (STAR v2.4.1d) aligner to the human genome (hg38)^18^. Quantification algorithms (featureCounts v1.4.6 (ref. 19) were applied using chromosomal location annotations for known miRNA (Mirbase v.21 (ref. 20), piRNA (piRNAbank v.2 (ref. 21), snoRNA (Ensembl v.84 (ref. 22), and snRNA (Ensembl v.84 (ref. 22) locations^12^. Detection of novel miRNA is performed using the miRDeep2 algorithm (v2.0.0.5), which considers the relative free energy of miRNAs and their random folding *P*-values^13^. Chromosomal position of expressed small RNAs was plotted against and hg38 karyotype obtained from UCSC Genome Browser (Fig. 2). According to OASIS sncRNA software recommendation (v2.0), sncRNA species were considered expressed if the total reads across all samples considered summed to ≥5 reads^12^. Data resulting from species quantification can be found in Data Citation 1.

### Normalization and quantification

**Figure 5:**
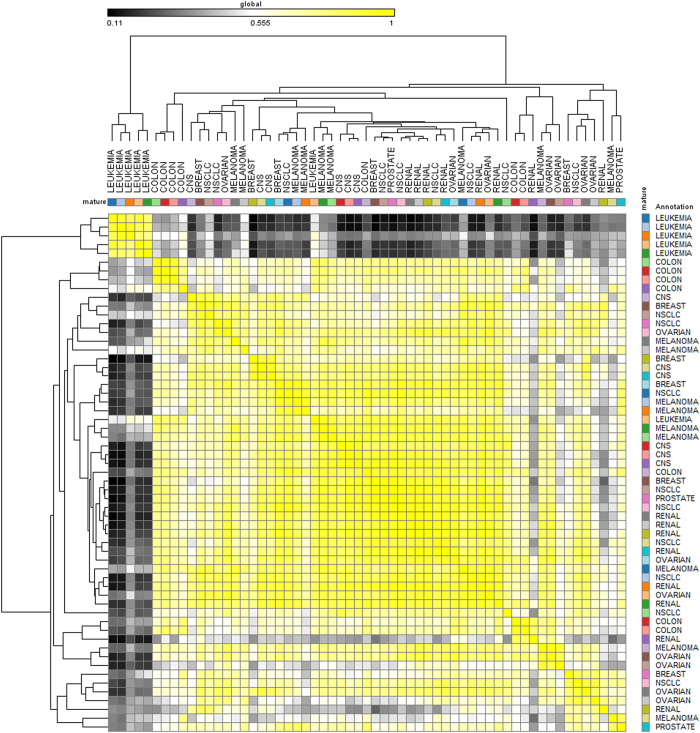


Raw reads were scaled/normalized using reads per kilobase exon per million mapped reads (RPKM) method^23^, and expression correlation matrices were created using Pearson scores with unsupervised hierarchal clustering performed using one-minus-Pearson correlation scores (Fig. 5). For validation of sncRNA expression, we then correlated miRNA species present in two published microarray cohorts of the NCI-60 cell lines. For the 50 (of the 59) cell lines also present in the Sanger Cell Line Database^24^ (http://www.cancerrxgene.org/translation/CellLine), raw reads from each unique sequence were correlated with expression of the sequence previously detected by microarray by rank-normalized Spearman`s correlations (Table 4 (available online only)), and performed a similar analysis against all cell lines present in the cohort described by Sokilde *et al.*^5^.

## Data Records

Raw unaligned sequencing reads (in FASTQ file format) are available through the *Sequence Read Archive* (Data Citation 2). Raw sequencing file names (in FASTQ format) are listed in Table 3 (available online only). A summary of raw sequencing reads for each detected small RNA species are available at through *Figshare* (Data Citation 1).

## Technical Validation

High-throughput sequencing allows for direct in-depth analyses of the human genome, recently revealing a critical role for the expression of the non-coding transcriptome in both genetic and epigenetic regulatory processes.

### Sequencing quality control

We examined only high-confidence reads from miRNA sequencing. Samples were sequenced to an average depth of 22.34±0.14 (mean±s.d.; Table 3 (available online only), Fig. 4b). In order to assure only the calling of high-quality sequencing reads, we filtered detected reads to only to include Phred scores ≥20. On average, samples had a Phred score of 33.24±1.28 (Table 3 (available online only), Fig. 4c). Additionally, reads for each sample had an average percent GC content of 46.26±1.6% (Table 3) (available online only). Unsupervised hierarchical clustering and similarity (one-minus-Spearman correlation) of normalized reads revealed relative similarity of sncRNA expression profiles across all cell lines and tissue types analyzed (Fig. 5).

### miRNA detection validation

In order to validate the detection of the sncRNA species in these cell lines, we correlated the raw reads per miRNA detected with corresponding miRNA detected by microarray^24,25^. This analysis was performed for the 50 NCI-60 cell lines present in the Sanger Cell Line miRNA Normalized Data from the Broad Institute (http://www.broadinstitute.org/cgi-bin/cancer/datasets.cgi; File name: Sanger_miR_data1.pn.cn.matlab2.res). Using Spearman’s Rank-Order correlation, we analyzed the correlation of this RMA-normalized miRNA expression to reads obtained from sequencing this cell line panel. Expression of miRNAs in all lines analyzed correlated significantly between sequencing and microarray analysis (Table 4 (available online only); *P*-values <0.0001, r_mean_=0.67). Similarly, we correlated sequencing-detected miRNA expression against a complete NCI-60 microarray cohort described by Sokilde *et al.*^5^. In this study, profiling was performed on the LNA-enhanced mercury Dx 9.2 microarray platform, and data was log_2_-normalized after pre-processing (Table 4; *P*-value range <0.0001–0.0647, r_mean_=0.28). Microarray data from multiple platforms was compared to sequencing data presented here in order to de-emphasize platform bias and illustrate the need for comprehensive profiling when considering small RNA expression^26^.

## Additional information

**How to cite this article:** Marshall, E. A. *et al.* Small non-coding RNA transcriptome of the NCI-60 cell line panel. *Sci. Data* 4:170157 doi: 10.1038/sdata.2017.157 (2017).

**Publisher’s note:** Springer Nature remains neutral with regard to jurisdictional claims in published maps and institutional affiliations.

## Supplementary Material



## Figures and Tables

**Table 1:** NCI-60 cell line characteristics.

**Tissue**	**Cell line**	**Subtype**	**Cell type of origin**	**Doubling time (hrs)**	**Year of origin**	**Culture conditions**
**BREAST**	BT-549	Ductal Carcinoma	Epithelial	*53.9*	1978	RPMI-1640+10% FBS
	HS-578T	Carcinoma	Epithelial	*53.8*	1977	DMEM+10% FBS
	MCF-7	Adenocarcinoma	Epithelial	*25.4*	1973	MEM+10% FBS
	MDA-MB-231	Adenocarcinoma	Epithelial	*41.9*	1974	L-15 Medium+10% FBS
	T-47D	Ductal Carcinoma	Epithelial	*45.5*	1981	DMEM+10% FBS
**CNS**	SF-539	Gliblastoma multiforme	Right Temporal Lobe	*35.4*	1986	MEM+10% FBS
	SF-295	Glioblastoma	Left Temporal Lobe	*29.5*	1986	RPMI-1640+10% FBS
	SF-268	Highly Anaplastic Astrocytoma	Right Parietal Lobe	*33.1*	1987	RPMI-1640+10% FBS
	U251	Glioblastoma	Non-Epithelial (CNS)	*23.8*	1978	EMEM+10% FBS
	SNB-75	Glioblastoma	Non-Epithelial (CNS)	*62.8*	1988	RPMI-1640+10% FBS
	SNB-19	Astrocytoma	Left parieto-occipital	*34.6*	1980	Ham's F10+10% FBS
**COLON**	COLO 205	Dukes' Type D, colorectal adenocarcinoma	Epithelial	*23.8*	1975	RPMI-1640+10% FBS
	HCT-15	Colon adenocarcinoma	Epithelial	*20.6*	1979	RPMI-1640+10% FBS
	HCC2998	Colon adenocarcinoma	Epithelial	*31.5*	1988	RPMI-1640+10% FBS
	HCT-116	Colon adenocarcinoma	Epithelial	*17.4*	1981	McCoy's 5a+10% FBS
	HT-29	Colon adenocarcinoma	Epithelial	*19.5*	1964	RPMI-1640+10% FBS
	KM12	Colon adenocarcinoma	Epithelial	*23.7*	1988	DMEM+10% FBS
	SW-620	Dukes' Type D, colorectal adenocarcinoma	Epithelial	*20.4*	1977	L15+10% FBS
**LEUKEMIA**	CCRF-CEM	Acute lymphoblastic leukemia	T lymphoblast	*26.7*	1964	RPMI-1640+10% FBS
	HL-60(TB)	Acutre promyelocytic leukemia	Promyeloblast	*28.6*	1977	IMDM+20% FBS
	K-562	Chronic myelogenous leukemia	Lymphoblast	*19.6*	1975	IMDM+20% FBS
	MOLT-4	Acute lymphoblastic leukemia	Lymphoblast	*27.9*	1980	RPMI-1640+10% FBS
	RPMI 8226	Plasmacytoma; myeloma	B Lymphocyte	*33.5*	1966	RPMI-1640+10% FBS
	SR	Large cell immunoblastic lymphoma	Lymphoblast	*28.7*	1983	RPMI-1640+10% FBS
**MELANOMA**	MALME-3M	Malignant melanoma	Fibroblast	*46.2*	1975	IMDM+20% FBS
	LOX-IMVI	Amelanotic melanoma	Skin	*20.5*	1988	RPMI-1640+10% FBS
	M14	Malignant melanoma	Skin	*26.3*	1976	RPMI-1640+10% FBS
	MDA-MB-435	Previously described as ductal carcinoma	Melanocyte	*25.8*	1976	L15+10% FBS
	SK-MEL-28	Cutaneous melanoma	Skin	*35.1*	1976	DMEM+10% FBS
	SK-MEL-5	Malignant melanoma	Stellate	*25.2*	1977	MEM+10% FBS
	SK-MEL-2	Malignant melanoma	Skin	*45.5*	1975	MEM+10% FBS
	UACC-257	Malignant melanoma	Skin	*38.5*	1991	RPMI-1640+10% FBS
	UACC-62	Melanotic melanoma	Skin	*31.3*	1991	RPMI-1640+10% FBS
**NSCLC**	A549	Adenocarcinoma	Epithelial	**22.9**	1972	RPMI-1640+10% FBS
	EKVX	Adenocarcinoma	Epithelial	*43.6*	1988	RPMI-1640+10% FBS
	HOP 92	Large cell carcinoma	Epithelial	*79.5*	1991	RPMI-1640+10% FBS
	HOP 62	Adenocarcinoma	Epithelial	*39*	1989	RPMI-1640+10% FBS
	NCI-H23	Adenocarcinoma	Epithelial	**33.4**	1980	RPMI-1640+10% FBS
	NCI-H322M	Small cell bronchioloalveolar carcinoma	Epithelial	*35.3*	1991	RPMI-1640+10% FBS
	NCI-H226	Squamous cell carcinoma; mesothelioma	Epithelial	*61*	1980	RPMI-1640+10% FBS
	NCI-H460	Carcinoma; large cell lung cancer	Epithelial	*17.8*	1982	RPMI-1640+10% FBS
	NCI-H522	Adenocarcinoma	Epithelial	*38.2*	1985	RPMI-1640+10% FBS
**OVARIAN**	IGR-OV1	Ovarian endometrioid adenocarcinoma	Endometreoid	*31*	1985	RPMI-1640+10% FBS
	NCI/ADR-RES	High Grade Ovarian Serous Adenocarcinoma	Epithelial	*34*	1986	RPMI-1640+20% FBS
	OVCAR-3	Adenocarcinoma	Epithelial	*34.7*	1982	RPMI-1640+10% FBS
	OVCAR-8	High Grade Ovarian Serous Adenocarcinoma	Epithelial	*26.4*	1984	RPMI-1640+10% FBS
	OVCAR-4	High Grade Ovarian Serous Adenocarcinoma	Epithelial	*41.4*	1984	RPMI-1640+10% FBS
	OVCAR-5	High Grade Ovarian Serous Adenocarcinoma	Epithelial	*48.8*	1984	RPMI-1640+10% FBS
	SK-OV-3	Adenocarcinoma	Epithelial	*48.7*	1973	RPMI-1640+10% FBS
**PROSTATE**	DU-145	Prostate carcinoma	Epithelial	*32.3*	1978	MEM+10% FBS
	PC-3	Adenocarcinoma	Epithelial	*27.1*	1980	F-12K+10% FBS
**RENAL**	A498	Carcinoma	Epithelial	*66.8*	1977	MEM+10% FBS
	CAKI-1	Clear cell renal cell carcinoma	Epithelial	*39*	1975	McCoy's 5a+10% FBS
	786-0	Renal cell adenocarcinoma	Epithelial	*22.4*	1976	RPMI-1640+10% FBS
	ACHN	Renal cell adenocarcinoma	Epithelial	*27.5*	1979	MEM+10% FBS
	RXF393	Renal cell carcinoma	Epithelial	*62.9*	1991	RPMI-1640+10% FBS
	SN12C	Renal cell carcinoma	Epithelial	*29.5*	1986	DMEM+10% FBS
	TK-10	Clear cell renal cell carcinoma	Epithelial	*51.3*	1987	RPMI-1640+10% FBS
	UO-31	Renal cell carcinoma	Epithelial	*41.7*	1991	DMEM+10% FBS

**Table 2:** Average number of sncRNA species detected and sequencing coverage per tissue type.

	**Number of sncRNA**							**Sequencing details**				
	**Total**	**miRNA**	**novel miRNA**	**piRNA**	**snoRNA**	**snRNA**	**Other**	**Average number of reads per sample**	**Average contig length**	**Avg. quality**	**%GC**	**Average coverage**
**Tissue type**	24,794	2,509	288	19,018	259	1,602	412	23,457,233	22.34	33.25	46.26%	28.94
Breast	11,079	1,905	183	6,909	239	1,248	403	25,310,156	22.32	33.23	45.81%	25.36
CNS	10,120	1,793	180	6,150	236	1,175	397	25,633,608	22.35	33.34	44.57%	45.08
Colon	13,985	1,977	211	8,050	232	1,276	392	25,850,349	22.22	33.2	46.15%	22.97
Leukemia	10,728	1,841	185	5,604	228	1,112	387	18,921,562	22.41	33.25	48.77%	20.23
Melanoma	9,536	1,830	179	4,694	224	1,020	383	16,116,671	22.36	32.64	46.56%	28.45
NSCLC	15,707	2,051	232	9,385	236	1,293	398	23,407,812	22.34	33.3	46.18%	22.8
Ovarian	10,422	1,916	188	6,312	238	1,167	398	27,221,870	22.4	34.12	46.56%	41.49
Prostate	6,167	1,393	121	2,589	216	771	344	35,637,053	22.3	33.54	44.44%	30.11
Renal	14,532	1,943	209	8,549	234	1,288	396	23,949,373	22.3	33	45.99%	27.05

**Table 3:** Sequencing quality metrics for sequenced cell line

**Tissue**	**External ID**	**Sample name (.fastq)**	**Total reads**	**Total alignments**	**Aligned**	**Total unaligned**	**Unaligned**	**Total unique**	**Unique**	**Total non-unique**	**Non-unique**	**Coverage**	**Avg. coverage depth**	**Avg. length**	**Avg. quality**	**%GC**
Breast	BT-549	MX1381-C9C07ANXX-1-CACTGT	26,779,757	20,087,593	74.84%	6,738,932	25.16%	19,994,122	74.66%	46,703	0.17%	0.62%	23.36	22.33	33.02	47.70%
	HS-578T	MX1383-C9C07ANXX-3-GGGGTT	24,961,809	20,875,168	83.42%	4,137,590	16.58%	20,773,358	83.22%	50,861	0.20%	0.35%	41.95	22.13	34.19	45.13%
	MCF-7	MX1384-C9C07ANXX-4-CTGGGT	43,741,142	32,170,069	73.27%	11,690,617	26.73%	31,931,263	73.00%	119,262	0.27%	0.81%	28.46	22.27	33.55	42.37%
	MDA-MB-231	MX1384-C9C07ANXX-4-GCCGGT	16,798,227	11,552,251	68.68%	5,261,551	31.32%	11,521,123	68.59%	15,553	0.09%	0.43%	19.43	22.45	33.25	46.01%
	T-47D	MX1387-C9C07ANXX-7-CTAAGG	14,269,847	9,004,918	63.04%	5,274,372	36.96%	8,986,041	62.97%	9,434	0.07%	0.48%	13.58	22.4	32.16	47.83%
CNS	SF-268	MX1386-C9C07ANXX-6-TAGTTG	35,537,775	32,273,096	90.47%	3,385,641	9.53%	32,031,463	90.13%	120,671	0.34%	0.28%	84.29	22.35	34.68	46.13%
	SF-295	MX1386-C9C07ANXX-6-CCGGTG	26,519,319	23,694,543	89.10%	2,890,215	10.90%	23,563,806	88.86%	65,298	0.25%	0.24%	70.66	22.39	34.43	42.31%
	SF-539	MX1386-C9C07ANXX-6-ATCGTG	25,500,558	23,012,418	90.00%	2,549,670	10.00%	22,889,448	89.76%	61,440	0.24%	0.25%	65.79	22.25	34.49	46.43%
	SNB-19	MX1387-C9C07ANXX-7-GCGTGG	32,717,872	21,567,743	65.75%	11,204,285	34.25%	21,459,531	65.59%	54,056	0.17%	0.85%	18.3	22.44	32.24	43.60%
	SNB-75	MX1387-C9C07ANXX-7-CATGGG	12,680,815	7,882,308	62.10%	4,805,816	37.90%	7,867,693	62.04%	7,306	0.06%	0.37%	15.13	22.2	32.05	45.20%
	U251	MX1387-C9C07ANXX-7-ATTCCG	20,845,308	13,006,365	62.30%	7,858,915	37.70%	12,966,435	62.20%	19,958	0.10%	0.58%	16.29	22.47	32.17	43.76%
Colon	COLO 205	MX1381-C9C07ANXX-1-TCAAGT	18,351,752	13,907,298	75.66%	4,466,918	24.34%	13,862,401	75.54%	22,433	0.12%	0.44%	22.77	22.23	33.19	45.58%
	HCC2998	MX1382-C9C07ANXX-2-GTAGCC	22,418,943	16,741,250	74.53%	5,710,380	25.47%	16,675,921	74.38%	32,642	0.15%	0.88%	13.4	21.83	32.83	47.12%
	HCT-116	MX1382-C9C07ANXX-2-TACAAG	19,321,624	13,822,479	71.42%	5,521,561	28.58%	13,777,662	71.31%	22,401	0.12%	0.54%	18.43	22.23	33.06	46.02%
	HCT-15	MX1382-C9C07ANXX-2-ATGTTT	22,372,577	15,816,346	70.57%	6,585,278	29.43%	15,758,281	70.44%	29,018	0.13%	0.62%	18.15	22.24	32.92	46.00%
	HT-29	MX1383-C9C07ANXX-3-CAAGTT	43,295,013	36,704,351	84.42%	6,746,834	15.58%	36,392,459	84.06%	155,720	0.36%	0.64%	40.79	22.23	34.19	45.58%
	KM12	MX1383-C9C07ANXX-3-GTCCTT	21,097,776	17,022,531	80.52%	4,108,873	19.48%	16,955,321	80.37%	33,582	0.16%	0.41%	29.68	22.35	34.03	46.33%
	SW-620	MX1387-C9C07ANXX-7-TTGCGG	34,094,755	22,349,352	65.38%	11,803,916	34.62%	22,232,420	65.21%	58,419	0.17%	0.92%	17.57	22.46	32.21	46.44%
Leukemia	CCRF-CEM	MX1381-C9C07ANXX-1-GATCTG	16,675,728	12,545,480	75.12%	4,148,470	24.88%	12,509,054	75.01%	18,204	0.11%	0.40%	22.55	22.48	33.4	49.05%
	HL-60(TB)	MX1382-C9C07ANXX-2-TGCTTT	14,448,374	9,588,877	66.29%	4,870,207	33.71%	9,567,467	66.22%	10,700	0.07%	0.51%	13.5	22.12	32.72	49.74%
	K-562	MX1383-C9C07ANXX-3-TCGCTT	35,495,593	28,500,403	80.03%	7,089,711	19.97%	28,311,603	79.76%	94,279	0.27%	0.60%	34.46	22.43	34.08	48.92%
	MOLT-4	MX1384-C9C07ANXX-4-GAGAGT	17,384,261	12,413,016	71.30%	4,989,141	28.70%	12,377,237	71.20%	17,883	0.10%	0.49%	18.25	22.49	33.61	48.27%
	RPMI 8226	MX1385-C9C07ANXX-5-AGGAAT	17,460,565	14,241,842	81.43%	3,242,340	18.57%	14,194,637	81.30%	23,588	0.14%	0.46%	22.37	22.47	34.06	48.91%
	SR	MX1387-C9C07ANXX-7-CCACTC	12,064,850	6,751,739	55.92%	5,318,416	44.08%	6,741,131	55.87%	5,303	0.04%	0.48%	10.23	22.48	31.62	47.70%
Melanoma	LOX-IMVI	MX1383-C9C07ANXX-3-CCTATT	31,657,831	25,924,121	81.64%	5,812,011	18.36%	25,767,653	81.39%	78,167	0.25%	0.47%	39.9	22.38	34.1	45.79%
	M14	MX1383-C9C07ANXX-3-GTTTGT	16,456,810	13,115,289	79.57%	3,361,434	20.43%	13,075,479	79.45%	19,897	0.12%	0.32%	29.6	22.45	34.08	47.29%
	MALME-3M	MX1383-C9C07ANXX-3-AGATGT	97,640	42,454	43.48%	55,187	56.52%	42,452	43.48%	1	0%	0.01%	4.85	22.33	29.76	46.02%
	MDA-MB-435	MX1384-C9C07ANXX-4-TATCGT	16,462,112	11,741,084	71.22%	4,736,983	28.78%	11,709,183	71.13%	15,946	0.10%	0.50%	16.9	22.36	33.4	47.73%
	SK-MEL-2	MX1386-C9C07ANXX-6-TGAGTG	30,951,870	27,402,647	88.25%	3,636,213	11.75%	27,228,840	87.97%	86,817	0.28%	0.32%	61.56	22.51	34.52	46.50%
	SK-MEL-28	MX1386-C9C07ANXX-6-CGCCTG	14,712,878	12,743,244	86.48%	1,988,450	13.52%	12,705,638	86.36%	18,790	0.13%	0.22%	41.57	22.26	34.24	46.57%
	SK-MEL-5	MX1386-C9C07ANXX-6-GCCATG	12,394,333	10,855,860	87.48%	1,552,173	12.52%	10,828,474	87.37%	13,686	0.11%	0.19%	41.26	22.5	34.4	46.38%
	UACC-257	MX1388-C9C07ANXX-8-AGCTAG	46,062	9,213	20.00%	36,849	80.00%	9,213	20.00%	0	0%	0%	2.13	22.17	26.55	46.63%
	UACC-62	MX1388-C9C07ANXX-8-GTATAG	22,270,501	15,834,593	70.97%	6,465,114	29.03%	15,776,225	70.84%	29,162	0.13%	0.62%	18.26	22.24	32.7	46.13%
NSCLC	A549	MX1381-C9C07ANXX-1-GCCTAA	24,542,337	18,533,593	75.35%	6,048,721	24.65%	18,453,696	75.19%	39,920	0.16%	0.53%	25.18	22.39	33.18	45.04%
	EKVX	MX1382-C9C07ANXX-2-AAGCTA	18,358,000	13,056,340	71.01%	5,321,501	28.99%	13,016,669	70.90%	19,830	0.11%	0.46%	20.08	22.13	32.99	45.71%
	HOP 62	MX1382-C9C07ANXX-2-GCATTT	22,874,592	16,227,434	70.81%	6,678,209	29.19%	16,165,375	70.67%	31,008	0.14%	0.58%	19.83	22.18	32.86	45.15%
	HOP 92	MX1382-C9C07ANXX-2-CGTACG	16,671,005	11,307,213	67.74%	5,378,661	32.26%	11,277,492	67.65%	14,852	0.09%	0.49%	16.63	22.16	32.7	45.80%
	NCI-H226	MX1384-C9C07ANXX-4-TCTTCT	18,745,702	12,999,215	69.24%	5,766,399	30.76%	12,959,420	69.13%	19,883	0.11%	0.52%	17.92	22.38	33.28	47.25%
	NCI-H23	MX1384-C9C07ANXX-4-CTATCT	19,234,429	13,677,426	71.00%	5,578,694	29.00%	13,634,066	70.88%	21,669	0.11%	0.51%	19.47	22.44	33.36	48.52%
	NCI-H322M	MX1384-C9C07ANXX-4-GATGCT	40,017,514	30,184,128	75.16%	9,938,560	24.84%	29,974,031	74.90%	104,923	0.26%	0.95%	22.98	22.47	33.58	45.76%
	NCI-H460	MX1385-C9C07ANXX-5-AGCGCT	12,712,247	9,871,928	77.57%	2,851,889	22.43%	9,848,794	77.47%	11,564	0.09%	0.32%	22.39	22.38	33.72	47.50%
	NCI-H522	MX1385-C9C07ANXX-5-CGGCCT	37,514,479	30,982,049	82.29%	6,643,642	17.71%	30,759,900	81.99%	110,937	0.30%	0.55%	40.76	22.53	34.02	44.85%
Ovarian	IGR-OV1	MX1383-C9C07ANXX-3-AGTCTT	28,718,340	23,886,771	82.95%	4,897,725	17.05%	23,754,573	82.72%	66,042	0.23%	0.42%	41.25	22.41	34.16	46.10%
	NCI/ADR-RES	MX1384-C9C07ANXX-4-ATCAGT	22,291,994	16,348,364	73.20%	5,974,630	26.80%	16,286,408	73.06%	30,956	0.14%	0.54%	21.99	22.37	33.49	47.02%
	OVCAR-3	MX1385-C9C07ANXX-5-AATTAT	29,981,152	25,036,208	83.26%	5,017,926	16.74%	24,890,371	83.02%	72,855	0.24%	0.46%	38.94	22.41	34.16	47.68%
	OVCAR-4	MX1385-C9C07ANXX-5-CCGTAT	28,168,179	22,957,608	81.29%	5,271,643	18.71%	22,835,575	81.07%	60,961	0.22%	0.43%	38.31	22.39	34.02	47.21%
	OVCAR-5	MX1385-C9C07ANXX-5-TAGGAT	24,989,662	20,898,933	83.43%	4,141,256	16.57%	20,797,961	83.23%	50,445	0.20%	0.40%	37.13	22.3	34.22	45.64%
	OVCAR-8	MX1385-C9C07ANXX-5-ATAGAT	21,340,411	17,484,019	81.76%	3,891,733	18.24%	17,413,389	81.60%	35,289	0.17%	0.35%	36.62	22.44	34.22	46.25%
	SK-OV-3	MX1386-C9C07ANXX-6-AAAATG	35,063,352	31,836,638	90.46%	3,344,843	9.54%	31,600,666	90.12%	117,843	0.34%	0.30%	76.21	22.49	34.54	46.04%
Prostate	DU-145	MX1382-C9C07ANXX-2-CTGATC	42,719,250	30,543,575	71.24%	12,284,018	28.76%	30,327,141	70.99%	108,091	0.25%	1.01%	21.68	22.17	32.93	42.80%
	PC-3	MX1385-C9C07ANXX-5-GCTCAT	28,554,855	23,674,991	82.68%	4,944,659	17.32%	23,545,544	82.46%	64,652	0.23%	0.44%	38.54	22.42	34.15	46.08%
Renal	786-0	MX1381-C9C07ANXX-1-CGTGAT	28,239,458	21,417,620	75.65%	6,875,229	24.35%	21,310,920	75.47%	53,309	0.19%	0.53%	28.79	22.32	33.21	45.18%
	A498	MX1381-C9C07ANXX-1-ACATCG	23,983,341	18,160,244	75.56%	5,861,378	24.44%	18,083,724	75.40%	38,239	0.16%	0.49%	26.57	22.26	33.14	44.19%
	ACHN	MX1381-C9C07ANXX-1-TGGTCA	26,253,335	20,447,816	77.70%	5,854,225	22.30%	20,350,495	77.52%	48,615	0.19%	0.50%	29.38	22.42	33.4	44.66%
	CAKI-1	MX1381-C9C07ANXX-1-ATTGGC	20,365,416	15,568,695	76.31%	4,825,051	23.69%	15,512,066	76.17%	28,299	0.14%	0.38%	29.39	22.37	33.42	44.53%
	RXF393	MX1386-C9C07ANXX-6-CTTTTG	29,863,988	26,677,423	89.05%	3,269,280	10.95%	26,512,156	88.78%	82,552	0.28%	0.34%	57.33	22.42	34.42	48.85%
	SN12C	MX1387-C9C07ANXX-7-TGTTGG	23,377,833	15,428,844	65.88%	7,976,649	34.12%	15,373,563	65.76%	27,621	0.12%	0.67%	16.45	22.28	32.21	45.57%
	TK-10	MX1387-C9C07ANXX-7-TTCTCG	26,774,556	17,534,744	65.36%	9,275,498	34.64%	17,463,407	65.22%	35,651	0.13%	1.16%	10.66	21.92	31.69	49.55%
	UO-31	MX1388-C9C07ANXX-8-TCTGAG	12,737,055	8,368,476	65.64%	4,376,740	34.36%	8,352,163	65.57%	8,152	0.06%	0.34%	17.82	22.44	32.53	45.42%
**MEAN**			**23,457,233**	**18,073,055**	**74.34%**	**5,429,370**	**25.66%**	**17,982,756**	**74.18%**	**45,107**	**0.16%**	**0.49%**	**29**	**22.34**	**33.25**	**46.26%**
**Standard Deviation**			**9,294,225**	**7,957,399**	**11.69%**	**2,519,478**	**11.69%**	**7,888,112**	**11.63%**	**35,871**	**0.08%**	**0.22%**	**17**	**0.14**	**1.28**	**1.64%**

**Table 4:** Spearman correlation of sequenced NCI-60 cell lines to published miRNA microarray expression levels

	**Broad**			**Sokilde** ***et al.***		
**Cell line**	**r**	**95% confidence interval**	**p (two-tailed)**	**r**	**95% confidence interval**	**p (two-tailed)**
786-0	0.7064	0.651–0.7543	<0.0001	0.3252	0.1976–0.4421	<0.0001
A598	n/a	n/a	n/a	0.2644	0.1327–0.3869	<0.0001
A549	0.6809	0.6218–0.7324	<0.0001	0.1815	0.04619–0.3103	0.0071
ACHN	0.6959	0.6389–0.7453	<0.0001	0.27	0.1387–0.392	<0.0001
BT-549	n/a	n/a	n/a	0.3202	0.1922–0.4376	<0.0001
CAKI-1	0.7004	0.6441–0.7492	<0.0001	0.1604	0.02453–0.2905	0.0175
CCRF-CEM	n/a	n/a	n/a	0.1251	-0.01157–0.2571	0.0647
COLO-205	0.6766	0.6169–0.7287	<0.0001	0.2974	0.1677–0.4169	<0.0001
DU-145	0.726	0.6735–0.7711	<0.0001	0.3293	0.2019–0.4457	<0.0001
EKVX	0.6248	0.558–0.6836	<0.0001	0.2634	0.1317–0.386	<0.0001
HCC2998	0.6512	0.5879–0.7066	<0.0001	0.3391	0.2125–0.4545	<0.0001
HCT-116	0.7019	0.6458–0.7505	<0.0001	0.3393	0.2127–0.4547	<0.0001
HCT-15	0.7193	0.6658–0.7654	<0.0001	0.2704	0.1391–0.3924	<0.0001
HL-60	0.6358	0.5704–0.6932	<0.0001	0.2533	0.1211–0.3767	0.0002
HOP-62	0.6899	0.632–0.7401	<0.0001	0.2566	0.1245–0.3798	0.0001
HOP-92	0.6576	0.5951–0.7121	<0.0001	0.2288	0.09537–0.3542	0.0006
HS-578-T	0.6283	0.5619–0.6867	<0.0001	0.2857	0.1554–0.4063	<0.0001
HT-29	0.6956	0.6386–0.7451	<0.0001	0.2875	0.1572–0.4079	<0.0001
IGR-OV1	0.6986	0.642–0.7476	<0.0001	0.3065	0.1774–0.4251	<0.0001
K-562	n/a	n/a	n/a	0.2657	0.1341–0.3881	<0.0001
KM12	n/a	n/a	n/a	0.3265	0.1989–0.4432	<0.0001
LOX-IMVI	0.6699	0.6091–0.7228	<0.0001	0.2637	0.132–0.3862	<0.0001
M14	0.6938	0.6365–0.7435	<0.0001	0.2585	0.1265–0.3815	0.0001
MALME-3M	0.5848	0.513–0.6485	<0.0001	0.3069	0.1779–0.4256	<0.0001
MCF-7	0.7235	0.6707–0.769	<0.0001	0.3271	0.1996–0.4438	<0.0001
MDA-MB-231	0.7125	0.658–0.7596	<0.0001	0.1837	0.04847–0.3123	0.0064
MDA-MB-435	0.7081	0.653–0.7558	<0.0001	0.2907	0.1607–0.4109	<0.0001
MOLT-4	0.7034	0.6476–0.7518	<0.0001	0.2809	0.1502–0.402	<0.0001
NCI/ADR-RES	n/a	n/a	n/a	0.3046	0.1754–0.4234	<0.0001
NCI-H226	0.6901	0.6323–0.7403	<0.0001	0.3613	0.2365–0.4743	<0.0001
NCI-H23	0.631	0.565–0.689	<0.0001	0.2649	0.1332–0.3873	<0.0001
NCI-H322M	n/a	n/a	n/a	0.3624	0.2378–0.4754	<0.0001
NCI-H460	0.6395	0.5746–0.6964	<0.0001	0.2794	0.1486–0.4006	<0.0001
NCI-H522	0.7047	0.649–0.7529	<0.0001	0.2856	0.1552–0.4062	<0.0001
OVCAR-3	0.6951	0.638–0.7446	<0.0001	0.3629	0.2383–0.4758	<0.0001
OVCAR-4	0.6731	0.6128–0.7256	<0.0001	0.3422	0.2158–0.4573	<0.0001
OVCAR-5	0.7101	0.6552–0.7575	<0.0001	0.2952	0.1655–0.415	<0.0001
OVCAR-8	0.6613	0.5994–0.7154	<0.0001	0.3407	0.2142–0.456	<0.0001
PC-3	0.6373	0.5721–0.6945	<0.0001	0.2893	0.1592–0.4096	<0.0001
RPMI-8226	0.6704	0.6098–0.7233	<0.0001	0.2641	0.1325–0.3866	<0.0001
RXF393	0.5656	0.4915–0.6316	<0.0001	0.3085	0.1797–0.427	<0.0001
SF-268	0.6788	0.6193–0.7305	<0.0001	0.2812	0.1505–0.4022	<0.0001
SF-295	0.6831	0.6243–0.7343	<0.0001	0.2986	0.1691–0.418	<0.0001
SF-539	0.6623	0.6005–0.7162	<0.0001	0.3037	0.1745–0.4227	<0.0001
SK-MEL-2	0.6294	0.5632–0.6876	<0.0001	0.2851	0.1546–0.4057	<0.0001
SK-MEL-28	0.6765	0.6167–0.7286	<0.0001	0.2867	0.1564–0.4072	<0.0001
SK-MEL-5	0.6095	0.5408–0.6702	<0.0001	0.3681	0.2439–0.4804	<0.0001
SK-OV-3	0.658	0.5956–0.7125	<0.0001	0.2613	0.1295–0.384	<0.0001
SN12C	0.7182	0.6645–0.7644	<0.0001	0.321	0.193–0.4382	<0.0001
SNB-19	0.6126	0.5441–0.6729	<0.0001	0.2332	0.09998–0.3583	0.0005
SNB-75	n/a	n/a	n/a	0.294	0.1642–0.4139	<0.0001
SR	n/a	n/a	n/a	0.1599	0.02397–0.29	0.0179
SW-620	0.7124	0.6578–0.7595	<0.0001	0.3805	0.2575–0.4915	<0.0001
T-47D	0.6827	0.6238–0.7339	<0.0001	0.3928	0.2709–0.5023	<0.0001
TK-10	0.7137	0.6593–0.7606	<0.0001	0.1981	0.06341–0.3258	0.0032
U-251	0.6242	0.5573–0.6831	<0.0001	0.2862	0.1558–0.4067	<0.0001
UACC-257	0.4873	0.4049–0.5618	<0.0001	0.2332	0.09994–0.3583	0.0005
UACC-62	0.676	0.6161–0.7281	<0.0001	0.3074	0.1785–0.426	<0.0001
UO-31	n/a	n/a	n/a	0.2975	0.1672–0.4175	<0.0001
